# Cover Plants-Mediated Suppression of Fusarium Wilt and Root-Knot Incidence of Cucumber is Associated With the Changes of Rhizosphere Fungal Microbiome Structure-Under Plastic Shed System of North China

**DOI:** 10.3389/fmicb.2022.697815

**Published:** 2022-04-04

**Authors:** Ahmad Ali, Ahmed S. Elrys, Liangliang Liu, Muhammad Iqbal, Jun Zhao, Xinqi Huang, Zucong Cai

**Affiliations:** ^1^School of Geography, Nanjing Normal University, Nanjing, China; ^2^College of Horticulture, Northwest A&F University, Yangling, China; ^3^Soil Science Department, Faculty of Agriculture, Zagazig University, Zagazig, Egypt; ^4^Jiangsu Engineering Research Center for Soil Utilization and Sustainable Agriculture, Nanjing, China; ^5^Institute of Soil Science, PMAS-Arid Agriculture University, Rawalpindi, Pakistan; ^6^State Key Laboratory Cultivation Base of Geographical Environment Evolution, Nanjing, China; ^7^Jiangsu Center for Collaborative Innovation in Geographical Information Resource Development and Application, Nanjing, China

**Keywords:** cover crops, ecological management, soil-borne pathogens, fusarium wilt, root-knot nematode incidence, fungal-mediated disease-suppression

## Abstract

Cover crops are known to alleviate the adverse effects of continuous cropping by influencing plant health and changing host fungal-microbiome structures. However, insight into the shift of rhizomicrobiota composition and their effects on plant growth performance and resistance mechanism is still limited under plastic shed cultivation (PSC). Four leafy vegetable rotations namely spinach rotation (SR), non-heading Chinese cabbage rotation (NCCR), coriander rotation (CR), and leafy lettuce rotation (LLR) were used as cover crops in 7-years of continuous cucumber planted soil (CC). Their ecological impacts were studied for plant growth performance, replant diseases incidence rate, and rhizosphere fungal microbiome. Compared to CC, SR showed a highly suppressive effect on fusarium wilt, i.e., by 13.2% in the spring season, while NCCR decreased the root-knot nematode incidence rate by 8.9% in the autumn season. Such protective effects caused a significant increase of shoot and fruit biomass and thus sustained the fruit quality of cucumber. High-throughput sequencing revealed that the CR, SR, and NCCR treatments altered the fungal community composition by increasing the abundance of the beneficial fungal genera, decreasing pathogenic taxa, and fostering the saprotrophic and symbiotic functions. However, the relative abundance of most of the potentially pathogenic fungal genera increased in CC and LLR cropping. There were 8 potential pathogens and 10 beneficial or biocontrol fungi characterized. It was found that *Paecilomyces*, *Chaetomium*, *Cladorrhinum*, *Zopfiella*, *Purpureocillium*, and *Metarhizium* were the putative biocontrol microbes that positively affected plant growth and replanted diseases inhibition. The characterized *Fusarium*, *Dactylonectria*, *Alternaria*, *Gibberella*, and *Aspergillus* were the key pathogenic fungal agents found to be negatively associated with plant growth characters, suggesting that rhizomicrobiome may play an important role in the occurrence of disease incidence of cucumber plants. Considering the ecological potential of some cover plants, this study suggested that rotation with spinach, non-heading Chinese cabbage, or coriander can enhance rhizosphere immunity by triggering the development of plant-protective fungal microbiomes under plastic shed cucumber cultivation.

## Introduction

Cucumber (*Cucumis sativus* L.), a popular and high economic cash crop all over the world, is commonly mono-cultured under plastic shed cultivation (PSC), especially in North China (Zhao et al., 2021; Zhou et al., 2021). However, intensively managed PSC soil with long-term continuous cropping has been reported to escalate soil acidification, salinization, nutrient leaching, and accumulation of autotoxic substances (Xin and Tao, 2020; Ni et al., 2021). Consequently, the vulnerable planting systems and their associated negative attributes are frequently reported for triggering the continuous cropping obstacles, i.e., a well-documented phenomenon of cucumber replant failure (Tian et al., 2011; Zhou and Wu, 2012; Zhao et al., 2017). Recently, drastic changes in rhizosphere microbiota structure by the build-up of soil-borne pathogens have also been proposed to contribute to this phenomenon due to cucumber monoculture (Zhao Y. et al., 2020; Liu and Zhang, 2021). As a result, cucumber monoculture is severely affected by soil-borne fungi fusarium wilt caused by *Fusarium oxysporum* f. sp. *cucumerinum* and plant-parasitic nematode (root-knot nematodes) caused by *Meloidogyne incognita* (Zakaria et al., 2013; Liu et al., 2016; Ye et al., 2020). These are the most destructive pathogens and often co-exist in the rhizosphere of cucumber monoculture, whereby, co-infection of *M. incognita* and *F. oxysporum* pertained to severe economic losses of up to 20–40% reduction in cucumber yield and fruit quality (Tian et al., 2011; Shi X. et al., 2019; Liu and Zhang, 2021). However, how rhizomicrobiome respond to the occurrence or suppression of soil-borne disease in the PSC soil is still not fully obvious. Therefore, understanding the influence of soil microbial communities is crucial for the sustainable development of a plastic-shed cultivation system for cucumber production.

The use of alternative rotation has been considered an effective method to alleviate the adverse effects of continuous cropping. Several studies have reported numerous soil benefits retunes by cover crop residues, including organic carbon (C) addition, soil nutrients cycling and soil structure stability (Berg and Smalla, 2009; Tian et al., 2011; Nouri et al., 2019). Further, the study of Adetunji et al. (2020) summarized the soil-plant productivity feedbacks of cover crops, including protection from weed colonization and growth, plant resilience to pathogens, increased crop yield, and reduced incidence and severity of aforementioned soil-borne pathogens. Cover cropping diversity can alter soil microbial structure by changing types and composition of exuded C substrates from roots (Andrew et al., 2016). Diverse cover crops significantly increase abundance and microbial diversity, which is an integral part of soil health and agro-ecosystem function (Lyu et al., 2020). Cover crops could be the mixture of heterogenetic plant materials based on the functional traits of individual cover crop species. Thus, improving soil microbial diversity by rotation may require specific crop combinations, which are expected to significantly impact soil microbial diversity. For example, greater microbial diversity and biomass were observed with celery rotation (Lyu et al., 2020) and Chinese cabbage–tomato rotations (Yang et al., 2020). The rotated soils compared to monocultured soil have been linked to higher soil organic matter (SOM), which plays a role in suppressing certain root rot and *Rhizoctonia* disease. Likewise, long-term field studies (10 years) suggested that different vegetable crops (onion or potato in rotations) affected the soil microbial communities. Greater microbial diversity imparts clear relationships to soil-borne disease incidence and to crop yields (Wright et al., 2015). At present, majority of studies have assessed the cover cropping effects mainly focused on bacterial communities and their specific microbial functions. However, there is still a paucity of information on how cover crops affect biological soil health through shifting soil microbiome structure, especially fungal communities.

It is well known that soil fungal communities are the first consumers of belowground inputs of plant-derived C and are more responsive than bacterial communities to vegetation cover and intensive agricultural management practices (Liu et al., 2019; Liu H. et al., 2020; Yao et al., 2020). Soil fungal microbiome structure governs the complex microbial communities colonizing plant roots and rhizospheric soils through diverse microbiota groups that play a decisive role in the agricultural ecosystem (Huang et al., 2020). Relative abundance and composition of core microbiome are known to be closely related to plant health and disease resistance of host plants (Mazzola, 2004). Certain rhizospheric fungal taxa are potential plant pathogens contributing to plant diseases, decreasing crop yield, or even leading to total crop loss (Lyu et al., 2020). Nevertheless, some endophytic fungal groups recruited plant-beneficial and disease-suppressive taxa that act as phytopathogens or biocontrol agents to inhibit or relieve plant diseases and stimulate plant growth (Yuan et al., 2018). It is of great significance to customize fungal-mediated microbiome structure and identify rhizosphere microbial traits and plant endophytes through high-throughput molecular methods, especially to reveal that which microorganisms are beneficial to plants, which are harmful to plants, and which are opportunistic pathogens (Du et al., 2020). Our knowledge of their wide range of key ecological functions, including saprotrophic, parasitism, symbiosis, and pathogenesis, is still in infancy under PSC (Han et al., 2019; Zhao et al., 2021).

Therefore, we used the high-throughput sequencing method and FUNGuild analysis to investigate the rhizosphere fungal composition and ecological functions during cover crops rotation and 7-year long-term continuous cucumber cropping systems in northwest China. The specific objectives of this research were to: (1) Determine the cover cropping effects on plant growth, fruit quality, and the suppression of Fusarium wilt and root-knot nematode incidences of cucumber plants, (2) estimate the changes in microbial community and characterize the rhizospheric fungal microbiome structure into beneficial and harmful fungal taxa, and (3) decipher the microbial mechanism of how conducive or antagonistic soil microbial shift in cropping system capacity may affect plant growth and diseases suppression of cucumber. We hypothesized that the diverse rotation with leafy vegetables provides greater suppression of cucumber diseases incidence than that under monoculture. However, this would be related to the changes in rhizosphere fungal community composition and diversity, and we supposed that some cover plants might have the potential to increase the relative abundance of disease suppressive taxa, correlating to Fusarium wilt and root-knot nematode suppression in cucumber plants.

## Materials and Methods

### Experimental Site and Soil Description

The field experiment was conducted in a typical 8-year-old commercial plastic shed from November 2016 to November 2017 in Yangling County, Shaanxi Province (34°17′N, 108°04′E). Continuous monoculture of cucumber and other vegetable crops are common and widely practiced in north-western parts of China under intensive PSC conditions. However, long-term cucumber monoculture in typical PSC production system (use of 6–14 times more N fertilizer and 2–7 times higher irrigation rates to double the cucumber yield) in North China (Xin and Tao, 2020; Ni et al., 2021) is more vulnerable to soil quality degradation and soil-borne pathogens invasion. This area is located on the south-central edge of the Loess Plateau, a semi-arid and humid area, with annual sunshine of 2,164 h and a frost-free period of 210 days. The length × width of the plastic shed structure was 60 m × 8 m and had a north-south orientation covered with a 0.2mm thick thermal polyethylene sheet. The mean air and soil temperatures were 23.3 and 19.4°C in the spring season and 17.3 and 16.1°C in the autumn season, respectively, under the plastic shed structure. The plastic shed soil was sandy loam alkaline, classified as Orthic Anthrosol cultivated for 7-years of continuous cucumber monoculture (double-cropping system from March 2009 to September 2016). The soil surface of the plastic greenhouse (0–20 cm) had SOM content of 15.59g kg^–1^, with a total nitrogen (N) of 1.43 g kg^–1^, total phosphorus (P) of 0.93 g kg^–1^, and a total potassium (K) of 7.15 g kg^–1^. The bulk density of the soil layer was 1.34 g cm^–3^, and the soil field capacity was 28.2%, while soil pH was 7.76. Groundwater with a nitrate concentration of 0.44 mg L^–1^ was used for irrigation. The high inorganic fertilizers were applied in previous consecutive cucumber production cycles, which caused critical ecological and environmental degradation.

### Treatments and Management

In total, five treatments with different cropping systems were compared: a conventional continuous cropping (control) and four cover crop treatments planted with leafy vegetables as rotations. The tested leafy species were commonly cultivated in the PSC system and include spinach (*Spinacia oleracea*), coriander (*Coriandrum sativum* L.), non-heading Chinese cabbage (*Brassica rapa* ssp. *pak choi*), and leafy lettuce (*Lactuca sativa* L.). Three replicates were maintained for each treatment in a complete randomized block design, and the size of each replicate plot was 3.6 m × 3.0 m. The leafy crops were generally raised during the off-season of cucumber (autumn-winter fallow period), planted in mid-November 2016, and harvested in the first week of February next year. Then, cucumber seedlings (*Cucumis sativus* L. cv. Jinglu No. 3) with two leaves were manually transplanted (0.6 m spacing between rows, 0.30 m between plants) from mid-February to June as the first growing season (winter-spring cultivation) and from August to October as the second growing season (autumn-winter cultivation) in 2017.

Seeds of all the leafy vegetables were equally broadcasted (27 kg ha^–1^) in each plot and managed according to recommended agronomic practices. No chemical fertilizers were added during cover crop plantation. However, each plot received a compound NPK fertilizer (16:16:8) at a rate of 300 kg ha^–1^ during the cucumber growth seasons. In the rotation experiment, there were five treatments: spinach rotation (SR), coriander rotation (CR), non-heading Chinese cabbage rotation (NCCR), and leafy lettuce rotation (LLR). The control was the continuous cropping cucumber (CC).

### Determination of Plant Growth and Fruit Quality Indicators

From spring to autumn of 2017, the morphological observations and fruit quality of cucumber plants were examined. To obtain the fresh shoot biomass (g), three plant samples were randomly taken from each replicate plot after cucumber was harvested, and the shoots above the cotyledon node were weighed using an electronic precision balance (0.001 g). Similarly, the plant samples (below the cotyledon node) were washed with tap water to remove the soil and then oven-dried at 70°C for 72 h to obtain the dry root biomass (g). Cucumber fruits were picked regularly at each harvesting stage following the conventional harvest practice. Small size or pulpy fruits were discarded, and average size fresh and immature fruits (25–30 cm long) were picked according to the commercial grading scale and weighed. The fresh weight of fruit biomass was an average of 60 plants in each plot.

The quality parameters of cucumber fruits were evaluated following the methods described by Zhao et al. (2017). Vitamin C content was measured using a titration method with 2,6-dichlorophenol. Soluble sugar contents were measured using the anthrone colorimetric method. Coomassie brilliant blue G-250 staining was used to measure the fruit soluble protein content.

### Assay of Diseases Incidence Index Measure

The Fusarium wilt and root-knot nematode are severe soil-borne diseases in the continuous monoculture of cucumber production. Thus, disease incidence rates were monitored under different cropping practices.

Fusarium wilt incidence was recorded at 30, 60, and 90 days after planting (DAP; spring season), and 30 and 60 DAP (autumn season) by counting the number of wilted plants. A total of 30 plants were randomly observed in each plot. The disease incidence index was based on observations, including foliage chlorosis, leaf wilt, and necrosis and visual scales (0 = no symptoms, 1 = light or moderate wilt with light vascular discoloration in the stem, 2 = severe wilt with vascular discoloration in the stem, and 3 = severally infected plants). The average disease incidence was expressed as the percentage of infected plants per total number of plants.

The root-knot indices were determined after cucumber harvest in both seasons. Usually, 10–15 plants per plot were randomly digged to determine the degree of root galling and root damage, recognizing six visual scales according to Taylor and Sasser (1978). The evaluation of root galling was according to the % scale 0–7, where 0 = no root galling, 1 = slight galling (1–25%), 3 = moderate galling (26–50%), 5 = heavy galling (51–75%), 7 = very severe galling (76–100%).

The disease index (DI) was calculated using the following formula: DI = [Σ (the number of diseased plants × disease severity index)/(7 × the number of plants rated) × 100].

### Soil Sampling

A total of 15 rhizospheric soil samples (five treatments × three replicates) were collected at the harvesting stage of cucumber after completing the two crop growth seasons. The composite soil samples were randomly collected from ten individual plants for each treatment, and rhizospheric soil sampling was done by following the method described by Lyu et al. (2020). Briefly, cucumber roots were carefully unearthed from the soil, and visible soil particles were gently shaken, and fresh roots of each cucumber plant were collected into a 50 mL centrifuge tube. Soil tightly adhered to the root surface was referred to as rhizospheric soil matrix. The collected rhizospheric soil was immediately transferred to the laboratory on ice and kept in a −80°C refrigerator for DNA extraction.

### DNA Extraction, PCR and Illumina HiSeq Sequencing

Microbial DNA was extracted using the E.Z.N.A. stool DNA Kit (Omega Bio-tek, Norcross, GA, United States) according to the manufacturer’s instructions. The concentration and purity of genomic DNA were determined using NanoDrop 2000 UV-Vis spectrophotometer (Thermo Scientific, Wilmington, DE, United States), and the DNA quality was confirmed using 2% agarose gels electrophoresis.

The ITS rRNA genes were amplified using the fungal-specific primers ITS3_KYO2F (5′-GATGAAGAACGYAGYRAA-3′) and ITS4R (5′-TCCTCCGCTTATTGATATGC-3′) (Bellemain et al., 2010) in a thermocycler PCR system (GeneAmp 9700, ABI, Foster, CA, United States). The PCR reaction was performed in triplicate 50 μL mixture containing 5 μL of 10× KOD Buffer, 5 μL of 2.5 mM dNTPs, 1.5 μL of each primer (5 μM), 1 μL of KOD Polymerase, and 100 ng of template DNA. The amplification program started with an initial denaturation at 95°C for 2min, followed by 27 cycles at 98°C for 10s, 62°C for 30 s, and 68°C for 30 s, and a final extension at 68°C for 10min. The PCR products were checked by 2% agarose gels and purified using the AxyPrep DNA Gel Extraction Kit (Axygen Biosciences, Union City, CA, United States) and quantified using QuantiFluor™ -ST (Promega, Madison, WI, United States).

Sequencing libraries were generated using TruSeq ^®^ DNA PCR-Free Sample Preparation Kit (Illumina, United States). The library quality was assessed on the Qubit^®^ 2.0 Fluorometer (Thermo Fisher Scientific, Hudson, NH, United States), and the purified amplicons were pooled in equimolar concentrations. Finally, the dual index sequencing of paired-end 250 bp was run on Illumina HiSeq2500 PE250 instrument (Illumina, San Diego, CA, United States).

### Processing of ITS Gene Sequenced Data

The raw reads of ITS rRNA gene were analyzed using the pipeline of quantitative insights into microbial ecology (QIIME) (version 1.9.1) (Caporaso et al., 2010). Paired-end reads were assembled using FLASH (fast length adjustment of short reads) software, and the low sequence reads with a length of less than 250 bp were removed for further analysis (Liu H. et al., 2020). The chimera sequences were filtered and discarded using the UCHIME algorithm (Edgar et al., 2011) with the UNITE fungal reference database (Koljalg et al., 2005). High-quality sequences were assigned and clustered into operational taxonomic units (OTUs) at a 97% similarity level using UPARSE pipeline^1^ (Edgar, 2013). Taxonomic assignment of each OTU was performed using Ribosomal Database Project (RDP) Classifier (Version 2.2) (Cole et al., 2014). The raw reads of fungal sequences were deposited into the NCBI Sequence Read Database under the accession number of BioProject PRJNA721529.

### Functional Prediction of Rhizosphere Fungal Community

FUNGuild database (Nguyen et al., 2016) was adopted to predict the ecological functions of ITS genes for fungi. FUNGuild analysis classified the fungal OTUs sequence into general ecological categories, identified as trophic modes with saprotrophs, pathotrophs, and symbiotrophs.

### Statistical Analysis

The microbial data of ITS fungal genes were statistically analyzed using the R packages (v. 1.12.0) and Vegan (version 2.5.5.5). Alpha-diversity indices (number of observed species, Chao, and a Shannon index) were calculated using QIIME. To show the abundance statistics of β-diversity, a species classification tree was constructed in a Perl script and visualized using SVG. Each taxon with a high abundance was selected to construct the phylogenetic tree of species. The fan-shaped pie chart components were drawn by combining the abundance information of species and the expression profile information in each sample. The different color regions in each pie chart represent the ratios of different samples on the taxon. The radius represents the proportion of the number of tags in the taxon to the total number of tags. The larger the radius is, the higher the abundance is. The UniFrac weighted distance matrix is used to generate principal coordinate analysis (PCoA map) both at genus and order level in order to further evaluate the sampling differences of community structures. UniFrac weighted non-metric multidimensional scaling (NMDS) analysis was drawn using the vegan package in R language to study the similarities and dissimilarities between different samples. A heatmap was generated to reveal the relative abundance pattern of top fungal genera using the heatmap package in the statistical program R. Spearman’s correlation coefficient test in R (Version 1.12) was computed to reveal the possible relationships between pathogenic and beneficial fungal taxa and plant growth characters and demonstrated in heatmap graph. Venn diagram (v 1.6.17) 10 package and UpSetR (v1.3.3) 11 package of R language were used for OTUs comparison of the common or unique OTUs among different samples. In addition, network analysis based on OTUs was performed in the R to reveal the interactions among fungal species (>0.1% relative abundance). Mantel test were performed using the vegan package in R for estimating the correlation among fungal community functions, plant growth attributes, and disease incidences.

Statistical analysis related to plant growth and disease incidence data were carried out with SPSS (18.0) following the One-way analysis of variance (ANOVA). The Least Significant Difference (LSD) test was used for multiple comparisons of significant differences (*P* ≤ 0.05).

## Results

### Cucumber Plant Growth, Diseases Incidence Suppression, and Fruit Quality

The experimental data indicated that different vegetables’ rotations affected the plant growth in both seasons (Table 1; *P* < 0.05). Compared to continuous cucumber cropping (CC), the SR (spinach rotation) and NCCR increased the fresh weight of cucumber shoot biomass by 22.9 and 32.1%, respectively, in spring 2017 and autumn 2017 (Table 1). Generally, the rotational treatments had no significant effect on dry root biomass relative to the CC treatment. However, the fresh weight of cucumber fruit biomass was significantly affected under the SR and NCCR rotations in both seasons. The resultant increase in fruit biomass production was 19.5 and 45.7%, respectively, compared to CC treatment. Surprisingly, 5.2% of fruit biomass was increased in the first growth season (spring 2017) but dramatically declined by 11.9% in the next growth season (autumn 2017) under LLR treatment (leafy lettuce rotation), as compared to CC treatment (Table 1; *P* < 0.05).

**TABLE 1:** Plant growth observations of cucumber under different cropping treatments (Means ± SE, *n* = 3).

Treatment	Spring-2017			Autumn-2017		
	Shoot biomass (f.w. g plant^–1^)	Root biomass (d.w g plant^–1^)	Fruit biomass (t ha^–1^)	Shoot biomass (f.w. g plant^–1^)	Root biomass (d.w g plant^–1^)	Fruit biomass (t ha^–1^)
CC	110 ± 2.87 b	1.09 ± 0.03 b	35.7 ± 2.08 b	95.2 ± 2.89 c	1.22 ± 0.07 a	3.26 ± 0.46 bc
SR	135 ± 1.64 a	2.21 ± 0.23 a	42.6 ± 3.18 a	124 ± 1.26 a	2.10 ± 0.53 a	3.44 ± 0.59 bc
CR	130 ± 3.58 a	2.15 ± 0.16 a	39.2 ± 3.36 ab	126 ± 1.43 a	2.10 ± 0.58 a	4.11 ± 0.17 ab
NCCR	132 ± 1.93 a	2.19 ± 0.14 a	39.4 ± 2.54 ab	129 ± 2.20 a	2.11 ± 0.15 a	4.75 ± 0.06 a
LLR	116 ± 3.02 b	1.23 ± 0.02 a	37.5 ± 0.79 ab	110 ± 1.91 b	1.19 ± 0.03 a	2.87 ± 0.23 c

*Values within the same column followed by different letters are significantly different at p < 0.05 according to the LSD test.*

*CC, continuous cucumber; SR, spinach rotation; CR, coriander rotation; NCCR, non-heading Chinese cabbage rotation; LLC, leafy lettuce rotation.*

**FIGURE 1:**
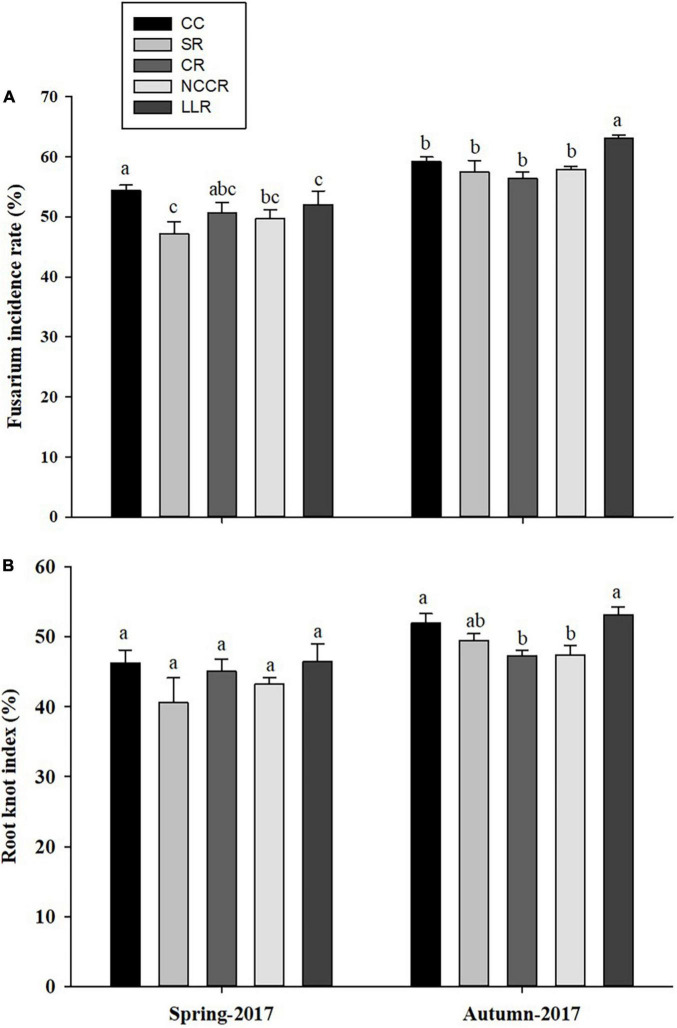
Effects of different cropping treatments on replant disease incidence (%) of cucumber plants during two growing seasons: *Fusarium wilt* incidences **(A)** and Root-knot index **(B)**. Different letters indicate significant differences in different treatments (*p* < 0.05). CC (continuous cucumber); SR (spinach rotation); CR (coriander rotation); NCCR (non-heading Chinese cabbage rotation); and LLC (leafy lettuce rotation).

The occurrence level of replanting diseases in cucumber plants is shown in Figures 1A,B. The results indicated that certain rotations effectively control the fusarium wilt incidence and root-knot nematode problem. The suppressive effect of SR, CR (coriander rotation), or NCCR rotations on fusarium wilt incidence rate was apparent compared to CC treatment. No differences were observed in the root-knot nematode index of cucumber plants grown in the spring season of 2017. Generally, SR treatments were found to be highly significant (*P* < 0.05) in their ability to reduce the fusarium wilt incidence (13.2%) in spring season 2017 when compared with other treatments (Figure 1A). Dramatically, LLR- induced fusarium wilt rate was slightly higher (2.34%) in the spring season of 2017, compared to CC. However, both CR and NCCR treatments significantly decreased the root-knot incidence rate to the next cucumber growing autumn season of 2017 (Figure 1B). Furthermore, it is obvious that cover cropping practices did not decrease the fruit quality of cucumber compared to the continuous cropping treatment (Table 2), but no significant differences (*P* < 0 .05) were found for short-term treatments incorporation.

**TABLE 2:** Cucumber fruits quality indices under different cropping treatments (Means ± SE, *n* = 3).

Treatments	Spring-2017			Autumn-2017		
	Vitamin C (mg kg^–1^)	Soluble protein (mg g^–1^)	Soluble sugar (%)	Vitamin C (mg kg^–1^)	Soluble protein (mg g^–1^)	Soluble sugar (%)
CC	118 ± 4.3a	1.40 ± 0.13a	2.03 ± 0.1a	117 ± 2.64a	1.30 ± 0.1a	2.10 ± 0.1a
SR	125 ± 2.6a	1.60 ± 0.17a	2.05 ± 0.02a	125 ± 1.32a	1.40 ± 0.1a	2.10 ± 0.1a
CR	119 ± 6.1a	1.50 ± 0.10a	2.05 ± 0.1a	121 ± 5.10a	1.30 ± 0.04a	2.10 ± 0.1a
NCCR	124 ± 3.4 a	1.50 ± 0.14a	2.06 ± 0.01a	126 ± 1.38a	1.40 ± 0.1a	2.10 ± 0.1a
LLR	120 ± 2.7a	1.50 ± 0.19a	2.05 ± 0.03a	119 ± 5.00a	1.30 ± 0.1a	2.10 ± 0.01a

*Values within the same column followed by different letters are significantly different at p < 0.05 according to LSD test. CC, continuous cucumber; SR, spinach rotation; CR, coriander rotation; NCCR, non-heading Chinese cabbage rotation; LLC, leafy lettuce rotation.*

### Diversity of Soil Fungal Communities

**FIGURE 2:**
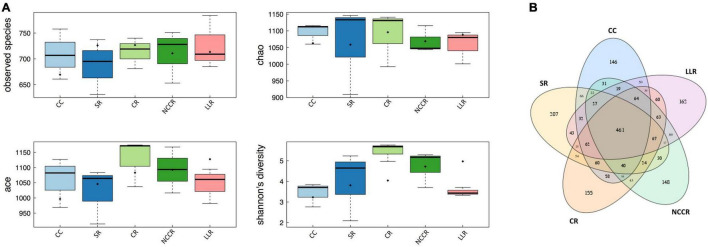
Boxplot and Venn diagram. **(A)** Boxplots showing alpha diversity (OTUs of observed species, Ace, Chao1 richness estimators, and Shannon diversity index) variation across different treatments. **(B)** The four-way Venn diagram indicates the OTUs of observed species, and the value in the overlapping circle represents the number of shared OTUs in the treatments.

The raw data obtained by the ITS sequencing of fungal community analysis contains 1,839,421 raw reads. A total of 1,788,639 high-quality sequences (effective ratio 88–95%) were extracted from all soil samples collected across the cropping system treatments (Supplementary Table 1). Among them, a mean number of total tags (119,242), Unique tags (31,155), Taxon tags (116,266), and Singleton tags (2,976) were counted to study the species diversity information of the samples (Supplementary Figure 1). The observed species and Chao index mainly reflect the number of OTUs in the sample. Our data revealed that observed species abundance and Chao diversity remained to be stable in response to all the treatment samples (Figure 2A). CR treatment-associated microbiota exhibited higher richness (ACE) than its continuous cucumber planted soil (CC). However, both CC and LLR treatments decreased the Shannon diversity index compared with SR, CR, and NCCR treatments. A total of 712 fungal OTUs were detected, with 461 common fungal OTUs identified in all the soil samples (Figure 2B).

### Community Composition and Relative Abundance of the Core Microbiota

**FIGURE 3:**
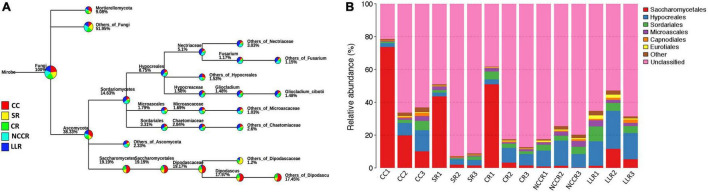
Phylogenetic tree and histogram exposed the dominant soil fungal communities. **(A)** The phylogenetic tree classification represents the relative abundance of fungal microbiota at all levels in treatments. **(B)** Histogram revealed the relative abundance of the top fungal orders in all soil samples under different treatments. CC1-CC3 represents samples from continuous cucumber cropping; SR1, SR2, and SR3 from spinach rotation; CR1-CR3 indicates samples from coriander rotation; NCCR1-NCCR3 from non-heading Chinese cabbage rotation, and LLR1-LLR3 from leafy lettuce rotation.

At the phylum level, *Ascomycota* and *Mortierellomycota* were the most dominant phyla, and their relative abundance (RAs) accounted for 38.33 and 9.08% of the detectable reads in the five treatment samples (Figure 3A). Majority of soil fungal microbiota were classified into six dominant fungal orders as *Saccharomycetales*, *Hypocreales*, *Sordariales*, *Microascales*, *Capnodiales*, and *Eurotiales* (Figure 3B). Among them, *Saccharomycetales* is the most abundant fungal order, which is significantly enriched in CC1 (73.68%), CR1 (51.04%), and SR1 (43.53%) groups. *Hypocreales* orders were mainly distributed in LLR rotation (range 14.89–23.29%), whereas the *Sordariales* and *Microascales* orders also showed significant differences to their RAs in the LLC group (Figure 3B). Both *Sordariales* and *Microascales* were accounted for 9.06 and 3.76%, respectively, of the detectable abundance in the LLR1 sample.

**FIGURE 4:**
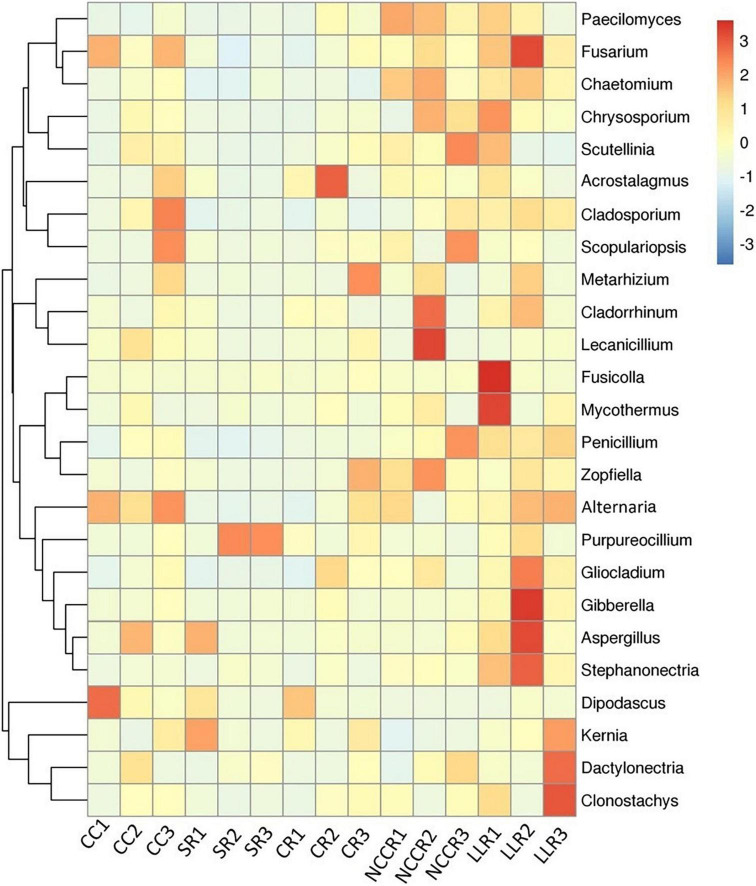
Heatmap of top 25 dominant genera of soil fungal communities in the different treatment groups. The color pattern of heatmap showing the relative abundance pattern of each taxon. The blue denotes low relative abundance, and the red symbolizes high relative abundance. The treatments abbreviation are defined in Figure 3.

We characterized the top 25 fungal taxa on the genus level, including some potential pathogenic and beneficial microbial species across all the soil samples, as shown in the heatmap abundance chart (Figure 4). The *Paecilomyces* taxa are significantly abundant under NCCR1 (0.21%) and NCCR2 (0.19%) groups. The keystone, beneficial taxa such as *Chaetomium*, *Cladorrhinum*, *Zopfiella, Purpureocillium*, and *Metarhizium*, were abundant in the rotation groups of NCCR2 (0.46%), NCCR2 (1.59%), NCCR2 (0.83%), SR3 (0.39%), and CR3 (0.45%), respectively. In potentially pathogenic genera, we also found their prevalent occurrence in both continuous cropping and rotation treatments. For example, *Fusarium* abundance in CC1-CC3 (0.25–0.46%), and LLR2 (2.77%), and *Alternaria* abundance in CC1-CC3 (0.47–0.74%) was significantly increased. *Gibberella*, *Aspergillus*, and *Dactylonectria* are also pathogenic, and they were predominant in the rotation group of LLR2 (3.46%), LLR2 (1.41%), and LLR3 (1.39%), respectively.

**FIGURE 5:**
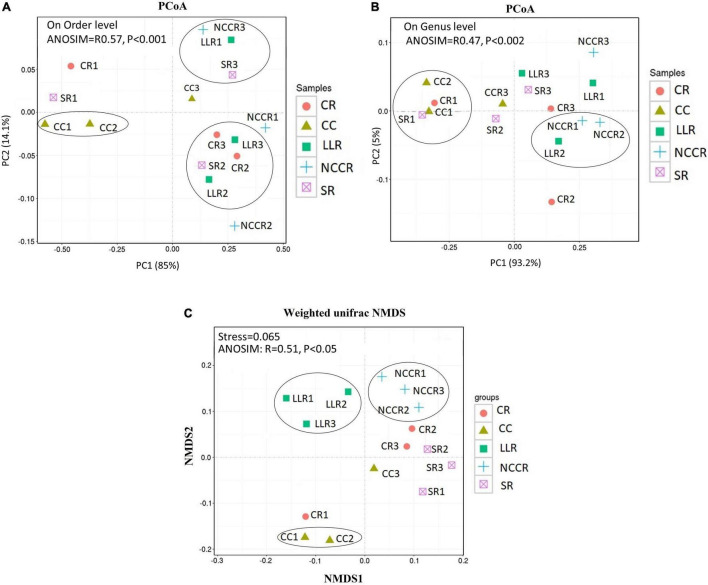
Variations of soil fungal communities across all the soil sample groups. Principal coordinates analysis (PCoA) showing the community differences at the order level **(A)** and the genus level **(B)**. UniFrac weighted non-metric multidimensional scaling (NMDS) ordination revealing community dissimilarities among different soil samples **(C)**.

The assemblage of β-diversity structure reflected the divergence response to the different cropping practices. Our study analyzed variations or similarities in fungal community structure that differed at both order and genus level (Figure 5). The variation of PCoA1 (85%) and PCoA2 (14.1%) axes divided the fungal communities of different sampling groups into four relatively independent parts (Figure 5A). CR3, CR2, LLR3, and LLR2 groups were distributed in the same part, indicating that the fungal communities in these groups were similar. In contrast, other groups were distributed in three parts, which indicated the community differences at the order level (Anosim test, *r* = 0.57, *p* = 0.001). The CC groups were distinctly separated from NCCR and LLR groups and reflected the genus level of community differences in these groups (Anosim test, *r* = 0.47, *p* = 0.002) (Figure 5B). Moreover, NMDS also explained the differences of fungal communities among different groups. The rotation groups of LLR and NCCR were separately clustered into other parts and separated from those of CC1, and CC2 groups (Figure 5C). Overall, NMDS also showed a significant difference in fungal composition between these three groups (Anosim test, *r* = 0.51, *p* = 0.05).

### Key Fungal Functional Groups

**FIGURE 6:**
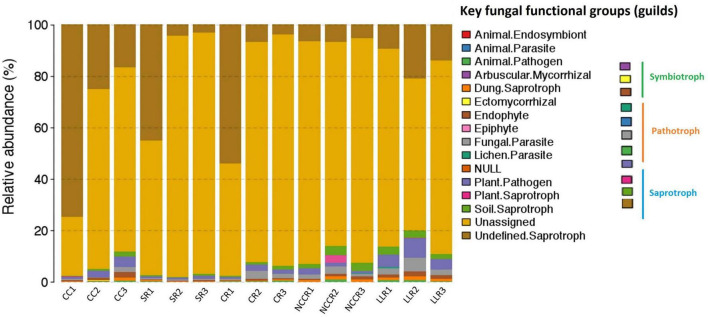
Relative abundance of fungal functional groups (guilds) inferred by FUNGuild in different treatments. CC1-CC3 represents samples from continuous cucumber cropping; SR1,SR2 and SR3 from spinach rotation; CR1-CR3 indicates samples from coriander rotation; NCCR1-NCCR3 from non-heading Chinese cabbage rotation, and LLR1-LLR3 from leafy lettuce rotation.

OTU richness of assigned fungal guilds revealed that 41, 34, and 27% of OTUs from all the treatment samples were identified to the trophic modes of saprotrophs, pathotrophs, and symbiotrophs, while the remainder were unassigned (Figure 6). Regarding the saprotrophic functional groups, wood saprotrophs, soil saprotroph, and plant saprotroph were the dominant trophic modes of LLR2 (0.67%), NCCR3 (0.28%), and NCCR2 (0.31%) soils. A significantly higher proportion of pathotrophs was identified in both continuous cropping soil and some rotation treatments. The high abundance of plant pathogens recognized as key fungal pathotroph was significantly increased in LLC (1.11–3.68%), CC3 (0.85%), and CC2 (0.38%), as seen in Figure 6. A fraction of fungal parasites in the LLC rotation (0.21–0.34%) was about twofold higher than those identified in the SR or CR rotations. Additionally, the proportion of symbiotrophs was significantly higher in SR rotations (0.29–0.39%) than CC treatments (0.05–0.09%).

### Relationships Between Aboveground Plant Indices and Soil Fungal Communities

**FIGURE 7:**
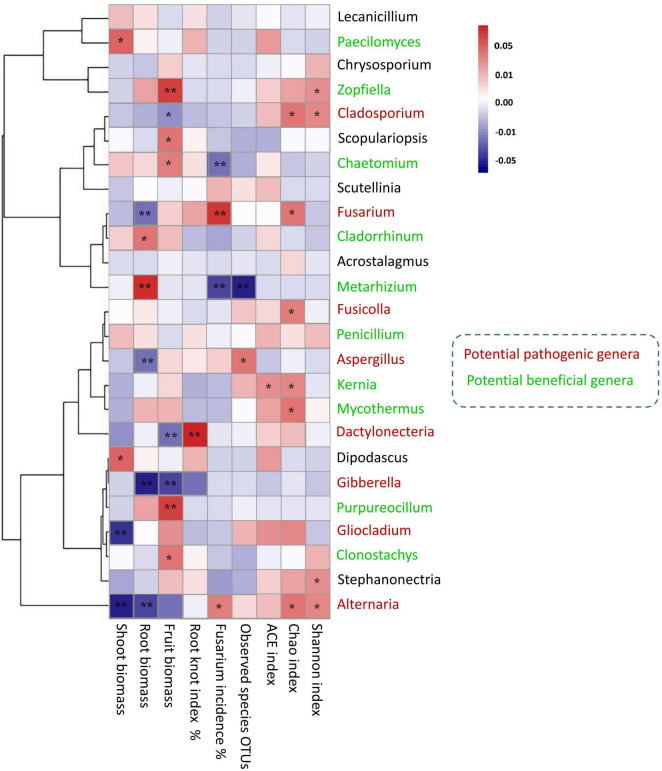
Heat map analysis illuminates the relationship between potential microbial taxa (beneficial and pathogenic) and plant growth characters and disease incidence. Correspondent Spearman’s rank correlation coefficient for each taxon suggests the degree of the positive and negative association at *0.05; **0.01 significance level.

Spearman’s correlation was used to connect the plant growth traits and disease response to the shift of potentially pathogenic and beneficial fungal communities (Figure 7). The genera of *Paecilomyces* and *Zopfiella* were positively correlated with shoot biomass (*r* = 0.61, *p < 0.05) and fruit biomass (*r* = 0.88, ^**^p < 0.01). The genus *Chaetomium* was positively correlated with fruit biomass (*r* = 0.65, *p < 0.05) and negatively correlated with fusarium wilt (*r* = −0.78, ^**^p < 0.01). Fusarium taxa had the positively correlated trend with fusarium wilt (*r* = 0.87, ^**^p < 0.01), and negatively correlated trend with root biomass (*r* = −0.76, ^**^p < 0.01). *Aspergillus* taxa negatively correlated with root biomass (*r* = −0.739, ^**^p < 0.01), whereas, *Dactylonectria* taxa was positively correlated with root-knot index (*r* = 0.89, ^**^p < 0.01). *Gibberella* taxa was found to be negatively associated with root biomass (*r* = −0.76, ^**^p < 0.01) and fruit biomass (*r* = −0.64, ^**^p < 0.01). *Alternaria* taxa was highly negatively correlated with shoot biomass (*r* = −0.80, ^**^p < 0.01) and root biomass (*r* = −0.75, ^**^p < 0.01). The diversity pattern of OTU species, Chao1 and Shannon, also shaped the overall abundance of soil fungal communities (Figure 7). Particularly, mantel test results revealed the functional compositions of soil fungal communities linked with plant growth and disease incidence. We found a significant (*p* < 0.02) positive correlation between the relative abundance of saprotrophic fungi and shoot biomass, whilst the abundance of symbiotrophic communities showed a significant (*p* < 0.004) negative correlation with fusarium wilt (Table 3).

**TABLE 3:** The Spearman’s correlation between fungal community functions and above-ground attributes determined by Mantel test.

Plant attributes	RAs^a^ of pathotrophic fungi		RAs of saprotrophic fungi		RAs of symbiotrophic fungi	
	*r*	*p*	*r*	*p*	*r*	*p*
Shoot biomass	0.169	0.134	**0.406**	**0.02**	0.28	0.07
Root biomass	−0.199	0.102	0.301	0.965	0.176	0.105
Fruit biomass	−0.186	0.9	0.046	0.562	0.257	0.061
Fusarium wilt	**0.616**	**0.001**	0.081	0.21	**−0.553**	**0.004**
Root-knot incidence	0.315	0.067	**−0.738**	**0.045**	0.115	0.193

*^a^The relative abundances of the fungal community assigned to the different functional roles using FUNGuild.*

Values in bold indicate significant correlations (***P < 0.001; **P < 0.01; *P < 0.05).

**FIGURE 8:**
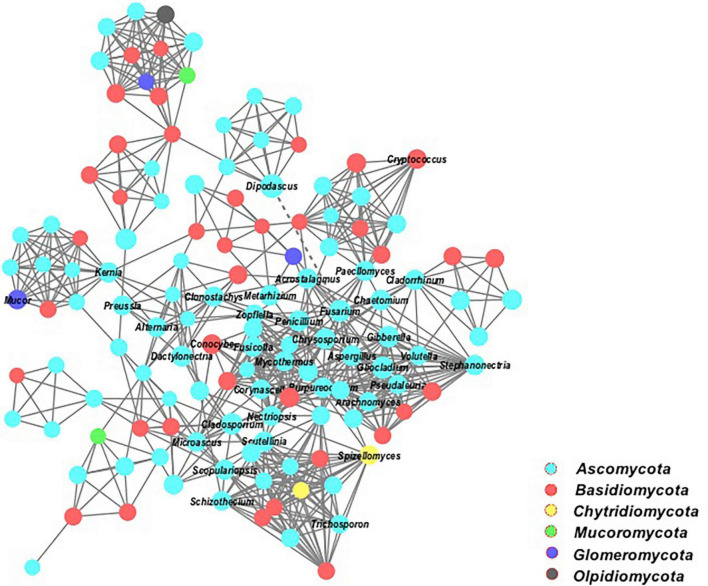
Co-occurrence network of the dominant fungal communities at genus level based on OTUs profiles. Each connection shows a spearman’s correlations (*p* < 0.05). Smooth straight lines connecting nodes represent positive (+), and dotted lines show negative (−) co-occurrence relationships.

In addition, the network analysis of this study explains the interactions of a microbial taxon in complex microbial communities (Figure 8). All the fungal species formed larger networks and were well connected under these cropping systems. The taxonomic composition of fungal community assemblages showed that most connections were positive. The keystone microbial members belonged to the phyla of *Ascomycota, Basidiomycota, Chytridiomycota*, and *Mucoromycota*.

## Discussion

### Plant Growth and Diseases Resistance Responses

Long-term continuously mono-cropped cucumber not only affect the plant growth but also causes serious fusarium wilt and root-knot nematode incidences (Tian et al., 2011; Liu et al., 2016). Therefore, our attention in the current study was to introduce alternative cover crop rotation for improving plant growth and reducing the severity of the diseases. The field experiment results indicated that rotation of spinach (SR), non-heading Chinese cabbage (NCCR), or coriander crop (CR) significantly enhanced the plant growth characters and controlled the disease incidence rate in both growing seasons. The results were in line with previous findings under PSCs, where cucumber fruit production increased by growing sweet corn as a summer catch crop (Guo et al., 2008), while disease incidence rate reduced after edible amaranth cropping rotation (Tian et al., 2011). Previous results also confirmed the exudation of sulfur-containing allelochemicals in the rhizosphere soil of celery under the cucumber/celery system, which could increase the cucumber fusarium wilt resistance (Gao et al., 2020). Furthermore, a significant increase in plant growth development and disease inhibition was achieved due to improved soil nutrient conditions. The SR treatment in the spring season and NCCR in the autumn season significantly increased the SOM, available N, and available P contents (Supplementary Table 2), and greater soil nutrient cycling under these crop rotations caused a substantial impact in terms of cucumber growth promotion and diseases suppressiveness (Supplementary Table 3). These results demonstrated that rotation of spinach and Chinese cabbage could play a better role to enhance the cucumber plant growth through soil nutrient accumulation (Lyu et al., 2020). However, cucumber plants are susceptible to replanted disease development under LLC (leafy lettuce rotation), as indicated by the severity of root-knot index and fusarium wilt. Such a contrary effect could reflect the poor competitive interaction among neighboring plants for resources and nutrients or be related to the difference in root exudates or plant-derived residual phytotoxicity (Berg and Smalla, 2009). Thus, our study emphasizes the importance of the careful selection of plant species and suggests the vulnerability of leafy lettuce to the host crop via their possible effects in soil nutrients decline (Supplementary Table 2) and may induce plant disease symptoms (Figures 1A,B).

### Soil Fungal Alpha Diversity Responses

Crop rotation did not significantly influence the species abundance and richness index (Figure 2A). This is inconsistent with those studies where soybean rotation or maize rotation have affected fungal diversity (Liu Z. et al., 2020). This difference depends on crop management practices, nutrient change, or environmental conditions, which may limit the influence of short-term cover cropping on soil fungal diversity. SR, CR, and NCCR rotations, however, restored the Shannon diversity compared to continuous cucumber cropping (CC). Long-term use of inorganic fertilizer, especially N induces the soil acidification in plastic shed soil (Han et al., 2015), and ultimately reduces the diversities of soil fungal biota under mono-cropped cucumber system (Zhao Y. et al., 2020). It is obvious that Shannon’s index of fungal diversity had a positively correlated trend with soil pH (Supplementary Table 4). The decrease of Shannon diversity in our continuous cucumber cropping soil may also reflect the similar concern followed by the local traditional practice of combining inorganic fertilizer and monoculture (Han et al., 2015; Zhao Y. et al., 2020).

### Fungal Community Structure and Their Ecological Role

Despite of similar growing conditions under plastic shed soil, phylogenetic diversity could primarily be shaped by heterogenetic plant biomass and root exudates (Martens, 2000). In our study, the assembly patterns of soil fungal communities in rotation treatments were more variable than the continuous cropping, especially at the genus level (Figure 5B, PCoA analysis). Similarly, we found that microbial structures under NCCR and LLR rotation also differed from each other and those of CC cropping (Figure 5C, NMDS analysis). Other studies also supported in parallel observations of divergent microbial structure, reporting that the aboveground plant community from diverse plant species has a greater determining effect over environmental factors in shaping the soil microbial assemblage (Garbeva et al., 2008; Schmidt et al., 2019). Moreover, CR and SR rotation were close together and assembled a similar pattern of soil fungal communities, explaining the consistency and stability of the core microbiome under plant cover soil (Andrew et al., 2016).

High-throughput ITS sequencing revealed that about 50% of the total fungal community was reshaped by *Ascomycota* and *Mortierellomycota* phyla’s dominant pattern. Both phyla have shown stronger environmental adaptability in the nutrient-rich plastic shed ecosystems (Liu C. et al., 2020). Ascomycetes are the key plant substrate decomposers that may have the ability to degrade the recalcitrant lignin-containing litter material in different soils (Almagro et al., 2021). *Mortierellomycota* phyla have been reported to play an ecological role in plant health improvement by generating the antagonistic substances, Arachidonic acid, an elicitor of phytoalexins in plants, suppressing plant disease (Deleón et al., 2018). Among the core family members, *Nectriaceae* was the dominant fungal family found abundantly in NCCR and LLR rotations, suggesting that these associated organisms may have some phytopathological significance (Hu et al., 2017). Similarly, fungal orders *Hypocreales* and *Sordariales* were dramatically more abundant under LLR treatment (Figure 3B), which have been reported with antifungal activities in previous reports (Shi M. et al., 2019; Huang et al., 2020). Some genera of *Sordariales* (*Mycothermus*) of this study can mobilize nutrients from organic substrates (Cai et al., 2003).

Cover cropping and monoculture systems altered the soil biota community that performs ecological functions ranging from saprotrophic to pathogenic and to symbiotic. FUNGuild revealed that soil saprotroph and plant saprotroph were the most abundant trophic mode of NCCC rotation. This suggests that these fungi are well adapted in NCCC soil environments and higher saprotrophic members likely reflect the greater extent and variety of plant root carbon availability with cover crops and are involved in organic matter decomposition, carbon cycling, and nutrient mobilization (Kramer et al., 2012). Plant pathogens mode was the critical pathotroph guild found in this study and significantly increased in both continuous cropping soil and LLC rotation. Pathotrophic fungi generally acquire nutrient substances from harmful host cells and negatively affect crop growth and productivity (Li et al., 2020). Consistent with our results, Niu et al. (2018) and Yao et al. (2020) also found a high plant pathogen abundance in the plastic shed-peeper rhizosphere and pea rotation, respectively, and collectively reported their subsequent diseases occurrence signals.

In contrast, SR rotations reflect the symbiotroph-enriched fungal community more than CC treatments. Interestingly, the increasing abundance of plant symbiotrophs fungi with spinach cover crop contributed to the suppression of fusarium wilt (Table 3), which is consistent with other observations of higher symbiotic partners (AMF, endophytes) associated with cover-cropped systems (Schmidt et al., 2019). These results imply that spinach species may drive greater ecosystem resilience since symbiotrophic functions under spinach cover cropping can vastly expand the surface area of plant roots, providing greater access of plants to nutrients and water in exchange for carbon (Wang et al., 2010; Raza et al., 2017).

### Fungal-Mediated Antagonistic Microbial Structure Promoted Growth Characters and Suppressed Disease Incidences

Rhizosphere microbiome directly influences the aboveground plant productivity under crop management practices (Franciska et al., 2017). The relative abundance of soil microbial community changed under different cropping environments, so their potential capabilities may also differ from plant to plant (Mazzola, 2004; Franciska et al., 2017). In this study, the abundance of *Chaetomium*, *Cladorrhinum*, *Zopfiella*, *Purpureocillium*, and *Metarhizium* was significantly increased in the rotation of NCCR, SR, and CR treatments compared to continuous cucumber cropping soil (Figure 4). Previous studies have also reported them as potentially beneficial and putative microbial species of the fungal microbiome (Huang et al., 2010; Li and Wu, 2018). The significant microbial shift indicates the potential of cover cropping to alter the fungal microbiome and to increase plant resistance. It has been hypothesized that plants actively recruit beneficial soil microorganisms in their rhizospheres, and interplay between plant and microbial communities has long been thought to be one of the key mechanisms that not only suppress soil-borne pathogens (Handelsman and Stabb, 1996) but also induce plant growth signals (Van Loon et al., 1998). Interestingly, these results were also confirmed by the Spearman correlation analysis in our research (Figure 7). We suggested that *Chaetomium* positively affect the fruit biomass (*r* = 0.65, *p < 0.05) and negatively correlated with fusarium wilt (*r* = −0.78, ^**^p < 0.01). In accordance with others, *Chaetomium* is a well-known bio-control agent to suppress FOC infection and is a putatively plant-beneficial fungal group (Kharwar et al., 2010). The *Cladorrhinum* niches were well adopted in higher soil nutrient conditions (Supplementary Table 5), and taxa significantly induced the healthy root biomass (Figure 7). Other reports also suggested a similar trend (Liu et al., 2019), indicating that *Cladorrhinum* taxa are the promising agents in the biocontrol of soil-borne pathogen of *Rhizoctonia solani*. The relative abundance of OTUs corresponding to the *Zopfiella* and *Purpureocillium* genera was stimulated in rhizosphere soil by SOM and available P addition (Supplementary Table 5). Both genera contain disease suppressive potentials against other hostile microbial species, for example, *Zopfiella* produces antibacterial compounds (*Zopfiellamides*) to inhibit the pathogenic bacterium (Daferner et al., 2002), whereas *Purpureocillium* is the biological control agent against plant-parasitic nematode (Wang et al., 2016). Both enriched taxa are significantly associated with promoting fruit biomass in this study (Figure 7). *Metarhizium*, known to insect-pathogenic fungi, is another putatively plant-beneficial fungal group. Their ability to excrete plant-derived antifungal compounds has been identified as a potential biological control agent to several crucifer insect pests (Song et al., 2017). The strong negative correlation between *Metarhizium* genus and fusarium incidence rate indicates the possible antagonistic effects in reducing plant disease or plant-parasitic insects in our soils (Figure 7). Fungal genera *Paecilomyces* positive for plant health were also promoted by increasing available K content, significantly improving shoot biomass production.

Furthermore, we found that long-term continuous cucumber cropping or some plant species led to a significant increase in the relative abundance of *Fusarium*, *Aspergillus*, *Gibberella*, *Alternaria*, and *Dactylonectria* (Figure 7). The higher abundances of these potentially pathogenic genera becomes the host-microbiome structure susceptible to adversely affecting plant growth and causing plant disease invasion (Zhao J. et al., 2020; Wang et al., 2021). Specifically, continuous cucumber cropping is more conducive to the abundance of *Fusarium* and *Alternaria*. The microbial abundance of these taxa may respond to environmental conditions, such as soil pH and the SOM (Supplementary Table 5), which negatively affect the shoot and root biomass (Figure 7). Other studies also characterized these taxa as potential pathogenic biomarkers from root exudates of continuous cucumber cropping (Zhou and Wu, 2012). *Fusarium* species contain various pathogenic microbes, which caused serious fusarium wilt infection in cucumber plants (Zhou and Wu, 2012; Chen et al., 2018), whereas *Alternaria* isolates caused early blight disease in tomato plants (Attia et al., 2020). Higher pathogen buildup in consecutive cucumber cropping may result in replant failure and reduction of yield and crop quality, a phenomenon described as soil sickness due to continuous cropping obstacles. However, rotation with SR, CR, and NCCR did not increase the abundance of pathogenic genera. We supposed that these cover cropping effects protected the cucumber rhizosphere ecology and enriched the disease-suppressive soils against pathogens assaults. By contrast, LLC rotation caused a significant increase in *Fusarium*, *Gibberella*, *Aspergillus*, and *Dactylonectria*. Higher abundance of these pathogenic genera was positively associated with plant disease index and negatively associated with plant growth indices. Notably, the higher relative abundance of genus *Dactylonectria* in leafy lettuce rotation may be related to negatively affecting fruit biomass and inducing root-knot incidence (Figure 7). Consistent with these results, recently, Liu et al. (2019) and Strom et al. (2020) confirmed that drastic shift of soil fungal communities associated with *Dactylonectria* pattern was a major contributor of the yield decline in corn-soybean rotations. They suggested that soil N accumulation and host-specific pathogens richness were to the limiting factors of yield decline. Results revealed that the soil fungal community networks have more correlated members and exhibited more positive links (Figure 8). Higher degree of cooperation or synergism within the fungal community after crop rotations was likely due to the addition of C source into the soil (Benitez et al., 2017).

In addition, we found a positive correlation among the diversity indexes (Chao and Shannon) of pathogenic genera (*Fusarium* and *Alternaria*) and plant diseases incidence rate (Figure 7). Based on our results, we propose that the increase in pathogenic microbial diversity could also induce aboveground plant infections (e.g., *Fusarium wilt* disease). Intriguingly, Huang et al. (2020) have provided evidence on the recruitment of pathogenic microbes in the *lisianthus* rhizosphere and root endosphere. They disclosed that the soil-borne pathogenic microbes could challenge the defense-related mechanism when the plant pathological condition was developed in the aboveground parts.

To our best knowledge, this is the first report that characterized the soil fungal microbiome structure into antagonistic and beneficial taxa under different plastic shed cucumber cropping systems. Although, the relative abundance pattern of some community members is important because they positively and antagonistically affect the above-ground cucumber growth. Future work should, however, require to manipulate the host fungal-microbiome structure through the potential plant species selection or plant composition, and decipher the beneficial and antagonistic microbial relationships in disease suppression and crop performance.

## Conclusion

Continuous cucumber cropping and leafy cover types affected plant health, aboveground growth characters, and rhizomicrobiome structure differently. Our results demonstrated that some potential cover crops are disease-resistant plant species and showed better plant growth improvement, disease inhibition, and conducive microbial assemblage. Generally, we observed that NCCR, coriander rotation or spinach rotation might have the most potent effects on the fungal microbiome assembly. Their recruited beneficial and antagonistic fungal taxa could provide some protective functions to host crop development and diseases inhibition. In contrast, fungal communities are more sensitive to cucumber monoculture and leafy lettuce plant species. The observed microbial structure and functions of both cropping systems were found almost parallel in disease manifestation and pathogenic microbial abundances. The study implication on plant diversity-assisted rhizosphere fungal microbiome structure and functions is critical for disease control, and soil-borne pathogen in a plastic shed cucumber system.

## Data Availability Statement

The datasets presented in this study can be found in online repositories. The names of the repository/repositories and accession number(s) can be found below: https://www.ncbi.nlm.nih.gov/, PRJNA721529.

## Author Contributions

AA: experimentation, data collection, data analysis, investigation, methodology, writing—original draft, visualization, and writing—review and editing. AE: visualization, software, and bioinformatics analysis. LL: visualization, data interpretation, and writing—review and editing. MI: bioinformatics analysis, review and editing. JZ: methodology, software, data curation, and investigation. XH: visualization, and writing—review and editing. ZC: conceptualization, project administration, supervision, funding acquisition, and writing—review and editing. All authors contributed to the article and approved the submitted version.

## Conflict of Interest

The authors declare that the research was conducted in the absence of any commercial or financial relationships that could be construed as a potential conflict of interest.

## Publisher’s Note

All claims expressed in this article are solely those of the authors and do not necessarily represent those of their affiliated organizations, or those of the publisher, the editors and the reviewers. Any product that may be evaluated in this article, or claim that may be made by its manufacturer, is not guaranteed or endorsed by the publisher.

## Footnotes

1
http://drive5.com/uparse/
