# Supplementary material for: Cover Plants-Mediated Suppression of Fusarium Wilt and Root-Knot Incidence of Cucumber is Associated With the Changes of Rhizosphere Fungal Microbiome Structure-Under Plastic Shed System of North China
Source: Front Microbiol. 2022 Apr 4;13:697815. doi: 10.3389/fmicb.2022.697815 (PMC9015784; doi:10.3389/fmicb.2022.697815)
Supplement: Supplementary file 1 [file Data_Sheet_1.docx]

**Cover plants-mediated suppression of fusarium wilt and root-knot incidence of cucumber is associated with the changes of rhizosphere fungal microbiome structure under plastic shed system of North China.**

Ahmad Ali **^1,2^** . Ahmed S. Elrys **^1,3^** . Liangliang Liu **^1,4^** . Muhammad Iqbal **^5^** . Jun Zhao **^1,4^** . Xinqi Huang **^1,4,6^**, Zucong Cai **^1,4,6,7^** ^*^

^1^ *School of Geography, Nanjing Normal University, Nanjing 210023, China*

^2^ *College of Horticulture, Northwest A&F University, Yangling, Shaanxi 712100, China*

^3^ *Soil Science Department, Faculty of Agriculture, Zagazig University, 44511, Zagazig, Egypt*

^4^ *Jiangsu Engineering Research Center for Soil Utilization & Sustainable Agriculture, Nanjing 210023, China*

*^5^Institute of Soil Science, PMAS-Arid Agriculture University, Rawalpindi-46300, Pakistan*

*^6^ State Key Laboratory Cultivation Base of Geographical Environment Evolution, Nanjing, Jiangsu Province 210023, China*

^7^ *Jiangsu Center for Collaborative Innovation in Geographical Information Resource Development and Application, Nanjing 210023, China*

*Corresponding Author: Zucong Cai

E-mail: [zccai@njnu.edu.cn](mailto:zccai@njnu.edu.cn)

Phone number: +8613057535534

**Total Tables: 5**

**Total Figures: 1**

**Table captions:**

**Table S1** Description of all identified sequences affiliated with soil samples using Illumina HiSeq analysis after experiments in 2017. CC1-CC3 represents samples from continuous cucumber cropping; SR1, SR2 and SR3 from spinach rotation; CR1-CR3 indicates samples from coriander rotation; NCCR1-NCCR3 from non-heading Chinese cabbage rotation, and LLR1-LLR3 from leafy lettuce rotation.

**Table S2** Soil properties after cucumber harvest under different cropping treatments (Means ±SE, n = 3)

**Table S3** Spearman’s correlation coefficients between plant growth and soil environmental variables

**Table S4** Spearman’s correlation coefficients between alpha diversity and soil environmental variables

**Table S5** Spearman’s correlation coefficients between soil properties and abundant fungal genera

**Figure caption:**

**Figure S1** The metagenomics analysis of ITS rRNA sequence profiling across all the soil samples. CC1-CC3 represents samples from continuous cucumber cropping; SR1, SR2 and SR3 from spinach rotation; CR1-CR3 indicates samples from coriander rotation; NCCR1-NCCR3 from non-heading Chinese cabbage rotation, and LLR1-LLR3 from leafy lettuce rotation.

| **Sample Name** | **Raw Tags** | **Clean Tags** | **Effective Tags** | **Max. length** | **Min length** | **Effective Ratio (%)** |
| --- | --- | --- | --- | --- | --- | --- |
| CC1 | 201837 | 189947 | 189947 | 449 | 202 | 91.45 |
| CC2 | 113885 | 111264 | 111264 | 449 | 202 | 89.45 |
| CC3 | 107961 | 106279 | 106279 | 449 | 201 | 91.45 |
| SR1 | 135915 | 131588 | 131588 | 449 | 201 | 91.57 |
| SR2 | 109645 | 107835 | 107835 | 449 | 204 | 89.56 |
| SR3 | 90767 | 90038 | 90038 | 449 | 201 | 90.72 |
| CR1 | 151951 | 147026 | 147026 | 449 | 203 | 91.95 |
| CR2 | 110964 | 108678 | 108678 | 449 | 206 | 90.19 |
| CR3 | 105225 | 104062 | 104062 | 449 | 208 | 90.28 |
| NCCR1 | 117129 | 115406 | 115406 | 449 | 202 | 91.31 |
| NCCR2 | 110523 | 106352 | 106352 | 449 | 202 | 88.36 |
| NCCR3 | 117070 | 114762 | 114762 | 449 | 201 | 89.97 |
| LLR1 | 111027 | 107131 | 107131 | 449 | 207 | 87.47 |
| LLR2 | 144073 | 139627 | 139627 | 449 | 204 | 90.63 |
| LLR3 | 111449 | 108644 | 108644 | 449 | 206 | 90.88 |

**Table S1**


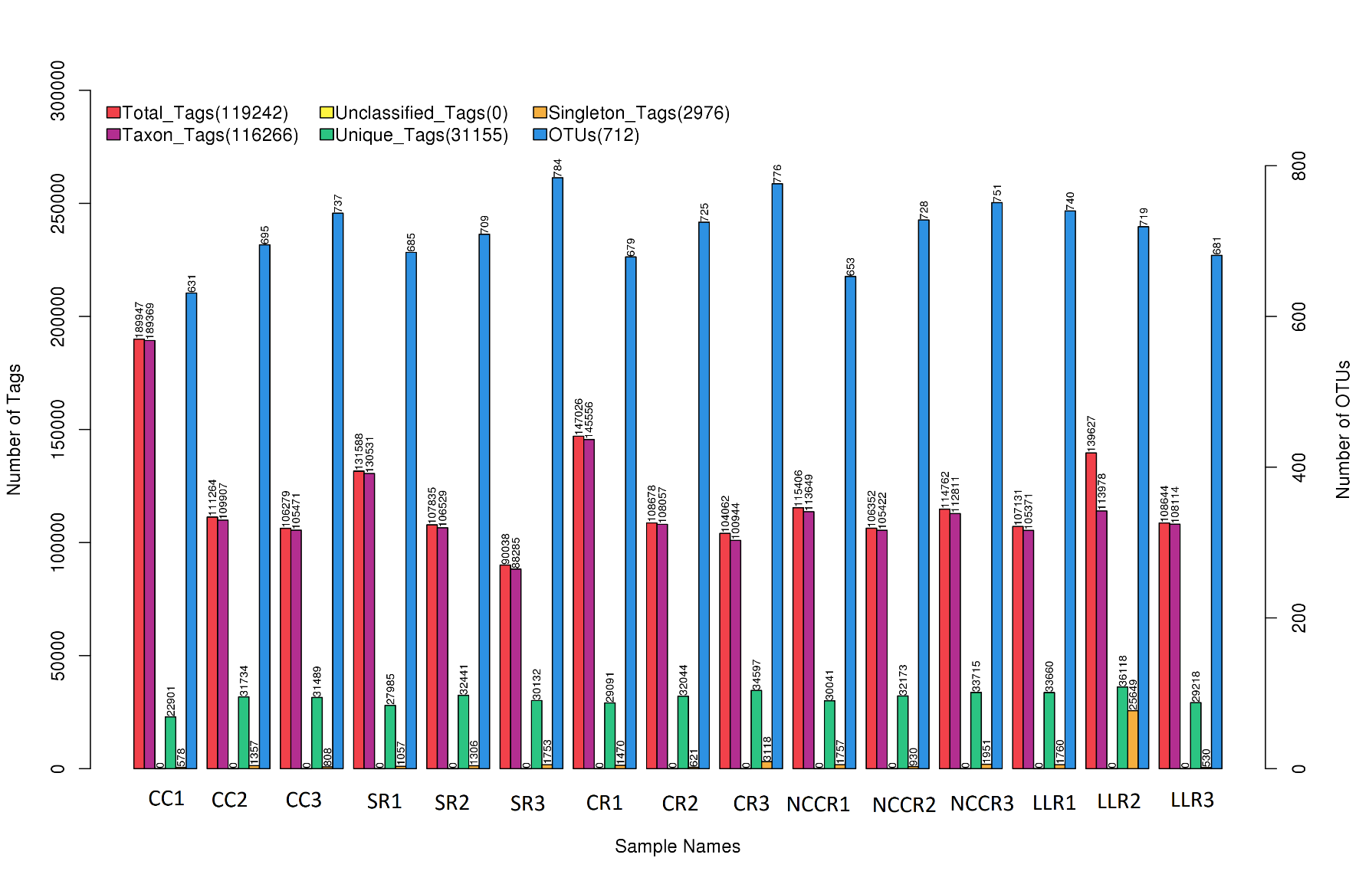


**Fig. S1**

**Table S2**

| Treatments | Spring-2017 | | | | |  | Autumn-2017 | | | | |
| --- | --- | --- | --- | --- | --- | --- | --- | --- | --- | --- | --- |
|  | pH | SOM  (g kg^-1^) | AN  (mg kg^-1^) | AP  (mg kg^-1^) | AK  (mg kg^-1^) |  | pH | SOM  (g kg^-1^) | AN  (mg kg^-1^) | AP  (mg kg^-1^) | AK  (mg kg^-1^) |
| CC | 7.74±0.12a | 18.63±1.28c | 122.8±3.03c | 64.39±2.00c | 345.4±1.75a |  | 7.73±0.12a | 21.18±1.58ab | 117.28±4.74b | 57.72±1.32b | 350.08±1.76cd |
| SR | 7.76±0.05a | 22.94±1.09a | 144.4±1.18a | 78.29±0.75a | 354.78±2.25a |  | 7.71±0.04a | 23.27±0.99ab | 136.2±5.43a | 65.81±2.60ab | 358.06±0.91bc |
| CR | 7.76±0.03a | 22.71±1.23a | 137.44±1.59ab | 73.25±2.25ab | 363.06±0.89a |  | 7.73±0.03a | 23.20±0.54ab | 138.9±1.48a | 69.94±2.00a | 365.84±1.79ab |
| NCCR | 7.71±0.05a | 21.55±1.29ab | 146.39±1.77a | 69.52±0.19bc | 359.89±1.85a |  | 7.70±0.02a | 24.72±1.00a | 142.16±1.67a | 72.39±0.91a | 368.58±2.01a |
| LLR | 7.73±0.03a | 19.45±1.59bc | 132.69±3.83b | 68.12±1.39bc | 351.49±3.82a |  | 7.73±0.04a | 20.09±0.42b | 123.89±0.37b | 61.53±5.03b | 347.69±5.40d |
| Values within the same column followed by different letters are significantly different at *P* < 0.05 according to LSD test. SOM: soil organic matter; AN: available soil nitrogen; AP: available soil phosphorus; AK: soil available potassium. CC (continuous cucumber); SR (spinach rotation); CR (coriander rotation); NCCR (non-heading Chinese cabbage rotation); LLC (leafy lettuce rotation). | | | | | | | | | | | |

| **Table S3** | | | | | |
| --- | --- | --- | --- | --- | --- |
| Plant growth characters | pH | SOM | AN | AP | AK |
| Shoot biomass | 0.669* | 0.689* | 0.764** | 0.529 | 0.688* |
| Root biomass | 0.447 | 0.494 | 0.233 | 0.257 | 0.654* |
| Fruit biomass | 0.555 | 0.559* | 0.719** | 0.659* | 0.586* |
| Fusarium incidence | −0.520 | −0.572* | 0.516 | −0.609* | −0.347 |
| Root knot index | 0.319 | −0.402 | 0.366 | −0.780** | −0.472 |
| SOM, soil organic matter; AN, available nitrogen; AP, available phosphorus; AK, available potassium.  * P < 0.05, ** P < 0.01. | | | | | |

| **Table S4** | | | | | |
| --- | --- | --- | --- | --- | --- |
| Plant growth characters | pH | SOM | AN | AP | AK |
| Observed species | 0.458 | 0.454 | 0.693* | 0.138 | 0.728* |
| ACE | −0.419 | 0.683* | −0.733* | −0.462 | 0.524 |
| Chao | 0.283 | 0.429 | 0.299 | 0.521 | 0.486 |
| Shannon index | 0.565* | 0.702 | −0.566* | −0.380 | −0.340 |
| SOM, soil organic matter; AN, available nitrogen; AP, available phosphorus; AK, available potassium.  * P < 0.05, ** P < 0.01. | | | | | |

| **Table S5** | | | | | | |
| --- | --- | --- | --- | --- | --- | --- |
| **Order** | **Genus** | **pH** | **SOM** | **AN** | **AP** | **AK** |
| *Hypocreales* | *Lecanicillium* | − 0.594* | 0.588* | 0.543 | 0.427 | 0.427 |
| *Eurotiales* | *Paecilomyces* | − 0.638* | 0.723** | 0.457 | 0.682* | 0.556* |
| *Onygenales* | *Chrysosporium* | 0.428 | 0.311 | 0.164 | 0.404 | −0.580* |
| *Sordariales* | *Zopfiella* | −0.611* | 0.832** | 0.606* | 0.834** | 0.368 |
| *Capnodiales* | *Cladosporium* | 0.676* | 0.647* | 0.589* | −0.528 | 0.719** |
| *Microascales* | *Scopulariopsis* | 0.539 | 0.428 | − 0.184 | 0.684* | − 0.427 |
| *Sordariales* | *Chaetomium* | − 0.940** | 0.597* | 0.685* | 0.589* | 0.585* |
| *Pezizales* | *Scutellinia* | − 0.595* | 0.410 | 0.196 | 0.555 | − 0.484 |
| *Hypocreales* | *Fusarium* | 0.485 | −0.720** | − 0.466 | − 0.595* | − 0.405 |
| *Sordariales* | *Cladorrhinum* | − 0.618* | 0.496 | 0.607* | 0.781** | 0.619* |
| *Hypocreales* | *Acrostalagmus* | 0.462 | 0.485 | 0.575* | 0.739** | − 0.532 |
| *Hypocreales* | *Metarhizium* | 0.465 | 0.635* | 0.796** | 0.599* | 0.247 |
| *Hypocreales* | *Fusicolla* | 0.212 | 0.457 | 0.666* | 0.653* | 0.129 |
| *Eurotiales* | *Penicillium* | − 0.699* | 0.528 | 0.731** | 0.621* | 0.349 |
| *Eurotiales* | *Aspergillus* | 0.529 | 0.465 | 0.484 | − 0.663* | − 0.477 |
| *Microascales* | *Kernia* | −0.316 | 0.805** | 0.318 | 0.471 | 0.295 |
| *Sordariales* | *Mycothermus* | 0.509 | 0.610* | 0.555 | 0.616* | 0.237 |
| *Hypocreales* | *Dactylonectria* | − 0.589* | 0.484 | −0.684* | − 0.227 | − 0.390 |
| *Saccharomycetales* | *Dipodascus* | −0.779** | − 0.697* | 0.499 | 0.744** | 0.692* |
| *Hypocreales* | *Gibberella* | 0.878** | − 0.655* | 0.563* | 0.873** | 0.730** |
| *Hypocreales* | *Purpureocillium* | 0.615* | 0.525 | 0.169 | 0.763** | 0.520 |
| *Hypocreales* | *Gliocladium* | 0.533 | 0.604* | 0.830** | 0.491 | 0.578* |
| *Hypocreales* | *Clonostachys* | 0.589* | 0.651* | 0.735** | 0.435 | 0.515 |
| *Pleosporales* | *Alternaria* | − 0.605* | − 0.619* | − 0.732** | 0.526 | − 0.373 |

SOM, soil organic matter; AN, available nitrogen; AP, available phosphorus; AK, available potassium.

* P < 0.05, ** P < 0.0
